# Maternal Weight Gain in Pregnancy and Risk of Obesity among Offspring: A Systematic Review

**DOI:** 10.1155/2014/524939

**Published:** 2014-10-02

**Authors:** Erica Y. Lau, Junxiu Liu, Edward Archer, Samantha M. McDonald, Jihong Liu

**Affiliations:** ^1^Department of Exercise Science, Arnold School of Public Health, University of South Carolina, Columbia, SC 29208, USA; ^2^Department of Epidemiology and Biostatistics, Arnold School of Public Health, University of South Carolina, 915 Greene Street, Room 459 Discovery Building, Columbia, SC 29208, USA; ^3^Nutrition Obesity Research Center, University of Alabama at Birmingham, Birmingham, AL 35233, USA

## Abstract

*Objectives*. To systematically review the evidence from prospective and retrospective cohort studies on the association between gestational weight gain (GWG) and offspring's body weight. *Methods*. Electronic databases PubMed, Web of Science, CINAHL, and Academic Search Premiere were searched from inception through March 18, 2013. Included studies (*n* = 23) were English articles that examined the independent associations of GWG with body mass index (BMI) and/or overweight status in the offspring aged 2 to 18.9 years. Two authors independently extracted the data and assessed methodological quality of the included studies. *Results*. Evidence from cohort studies supports that total GWG and exceeding the Institute of Medicine maternal weight gain recommendation were associated with higher BMI *z*-score and elevated risk of overweight or obesity in offspring. The evidence of high rate of GWG during early- and mid-pregnancy is suggestive. Additionally, the evidence on inadequate GWG and net GWG in relation to body weight outcomes in offspring is insufficient to draw conclusions. *Conclusions*. These findings suggest that GWG is a potential risk factor for childhood obesity. However, findings should be interpreted with caution due to measurement issues of GWG and potential confounding effects of shared familial characteristics (i.e., genetics and maternal and child's lifestyle factors).

## 1. Introduction 

Childhood obesity is a pandemic [1]. Over 155 million children aged 5–17 are overweight or obese worldwide [2]. In the United States, 16.9% children and adolescents aged 2–19 years are obese [3], while, in Europe, 12–36% children aged 7–11 years are overweight or obese. The childhood obesity epidemic has become a public health priority because of its immediate health consequences for children such as increased risk of type 2 diabetes mellitus and heart diseases [4, 5] and its long-term health impact such as increased risks of cardiovascular diseases, cancers and all-cause mortality in adulthood [6–8].

To reverse the obesity epidemic among children, identifying risk factors for prevention is crucial. Obesity is a result of individuals consuming more energy than they expend [9]. This positive energy balance is subject to multiple factors such as genetics, environment, and lifestyle factors [10–12]. In recent years, a growing body of literature suggests that intrauterine environment may also have a profound influence on the development of obesity later in life [13, 14]. One possible mechanism is that a suboptimal intrauterine nutritional environment that may modulate child's energy balance system through altering the developmental programming of appetite control and the metabolism of adiposity and adipocytes in fetuses. Children with the modified energy balance systems may be more vulnerable to obesogenic environment and thus increasing their risk of developing obesity in childhood [13, 14].

Maternal gestational weight gain (GWG), defined as the amount of weight a pregnant woman gained between the time of conception and the onset of labor [15], is one of the key markers of intrauterine nutritional environment. Between 1997 and 2007, approximately 46% of the pregnant US women gained more weight than the Institute of Medicine (IOM) recommendation [16, 17].

In recent years, this health issue has attracted an increasing number of researchers due to the potential impact of GWG on offspring's body weight in childhood [16–18]. Therefore, the objective of this review was to systematically summarize current knowledge regarding the association between GWG and offspring body weight in children aged 2 to 18.9 years from observational studies.

## 2. Materials and Methods

### 2.1. Search Strategy

A systematic review of existing cohort studies (prospective and retrospective) was performed following the PRISMA (preferred reporting items for systematic reviews and meta-analysis) statement [19] (see Supplementary Table 1 available online at http://dx.doi.org/10.1155/2014/524939) and the MOOSE (meta-analysis of observational studies in epidemiology) [20] guidelines. One author (EYL) conducted an electronic database search to retrieve English articles from PubMed, Web of Science, CINAHL, and Academic Search Premiere published from inception to March 18, 2013. The search strategies combined “gestational weight gain” or “pregnancy” or “maternal weight gain” with any of the following terms: outcomes (overweight, obesity, adiposity, or body mass index), target population (child, adolescent, offspring), and study design (longitudinal studies, cohort studies, or follow-up studies). Full electronic search strategies were described in Supplementary Table 2. To attain additional eligible articles, experts in the field were contacted; reference lists of located studies and relevant reviews [21, 22] were scanned. The search was limited to English articles published in international peer-reviewed journals. Book chapters, abstracts of conference proceeding, and dissertations were excluded.

### 2.2. Selection of Studies

To be included, articles had to (1) employ a cohort study design (prospective and retrospective), (2) focus on children aged 2 to 18.9 years, and (3) use GWG as an exposure and child age-and-gender specific BMI or overweight status used as an outcome. The current review focused on studies conducted in children and adolescents aged 2 to 18.9 years because the BMI-for-age percentiles from the Centers for Disease Control and Disease Prevention (CDC) and the International Obesity Task Force (IOTF) all start at age 2. BMI-for-age and overweight status were selected as the primary outcomes of interest because they were widely used in existing studies. Fat mass or waist circumference was not chosen because very few studies focused on these outcomes [23–25]. Studies were excluded if they focused on GWG in relation to child birth weight [26–28] or if the studies examined maternal prepregnancy overweight status rather than GWG in relation to offspring's body composition outcomes [29, 30].

The results from each database search and hand search were entered into Endnote database (Endnote X6, Thomas Reuters, 2012) and duplicated studies were removed. The title and abstract of the remaining studies were screened to identify potential articles for independent assessment of eligibility by two authors (EYL, JXL) and checked by the third author (JHL). Any disagreements were resolved by discussion among authors.

### 2.3. Data Analysis

The following data were extracted into a summary table by one author (EYL) and checked by another author (JXL): source (year of publication and country in which study was conducted); study characteristics (sample size, time period of the cohort, and child age at follow-up); GWG and child body weight measurements; confounders adjusted; and main findings. We decided not to use formal meta-analytic methods to estimate the effect of the exposure because of the expected heterogeneity in included studies, such as variations in exposure measures, length of follow-up, study population, and analytical methods. Therefore, the present review provided a qualitative evaluation of the longitudinal association between weight gain during pregnancy and child body weight outcomes. When a study presented results of multivariable statistical models, we summarized the findings based on the fully adjusted models. Discrepancies in data extraction were resolved by consensus of all authors.

#### 2.3.1. Methodological Quality Assessment

Two authors (EYL and JXL) independently rated the quality of included studies using an 8-item quality assessment checklist based on a published scale [15]. The quality of each study was graded as high, medium, or low on each of the following domains: background and objective, sample selection, specification of exposure, specification of outcome, data source, follow-up, comparability of analysis, analysis of outcome, and result interpretations.

## 3. Results

### 3.1. Summary of the Search

**Figure 1:**
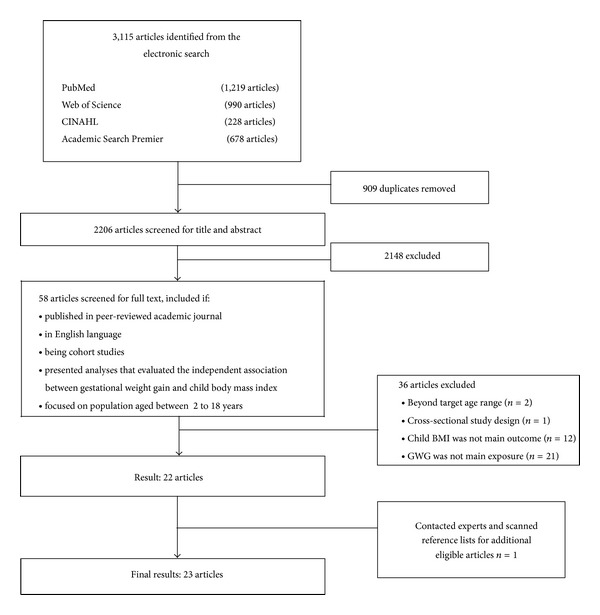
Flow diagram of study selection process.

The literature search yielded 2,869 hits. After eliminating 909 duplicates, 2,206 articles were screened by titles and abstracts. An additional 2,148 articles were excluded for not meeting our inclusion criteria. The remaining eligible full-texts articles (*n* = 58) were carefully reviewed and 38 of these articles were excluded due to (1) inclusion of samples outside targeted age range (*n* = 2), (2) not using a cohort study design (*n* = 1), (3) not using child BMI or overweight status as an outcome (*n* = 12), and (4) not using GWG as an exposure (*n* = 21). As a final step, contacting expert in the field and screening reference lists of eligible studies (*n* = 18) yielded an additional article [31]. Thus, a total of 23 articles [23–25, 31–50] were included in the systematic review (Figure 1).

### 3.2. Characteristics of Included Studies

Study characteristics are presented in Table 1. Fifteen studies [23–25, 32, 33, 35, 39–45, 47, 50] were based on a pregnancy cohort in which pregnant women were recruited during pregnancy and their offspring were followed prospectively during the childhood [51]. Six studies [34, 36–38, 46, 49] used mixed prospective cohort designs, in which maternal GWG was ascertained from medical records, and child's body weight was collected during the follow-up [51]. Three studies [31, 43, 48] used a retrospective design, in which maternal GWG was obtained from past records and data on child's body weight outcomes were either retrieved from medical record or ascertained at the time the study began [51].

Nine out of the 19 studies used data from historic cohorts (i.e., cohorts initiated between 1959 and 1990) [24, 33, 37–39, 43, 44, 46, 47]. Sixteen studies were conducted in the US [25, 31, 33, 35, 36, 38–43, 46–50] and seven studies in Europe [23, 24, 32, 34, 37, 44, 45]. Most of these studies included a reasonably large sample size (*n* ≥ 1000) with three exceptions (*n* < 700) [42, 43, 49]. Studies conducted in Europe and the US mainly enrolled Caucasian women; all but two [37, 46] enrolled both male and female offspring. Twelve studies focused on younger children (aged 3 to 5 years) [25, 33–36, 39, 40, 42, 43, 45, 48, 50], four studies on older children (aged 6 to 12 years) [23, 32, 46, 47], and three studies concentrated on adolescents (aged 13 to 18 years) [24, 37, 41]. Three studies examined the association of interest across age groups [31, 38, 44].

Three articles came from the Project Viva [25, 35, 40] and were treated as separate studies because they examined different GWG exposures. Two studies [33, 47] drew data from the National Collaborative Perinatal Project and were both included as separate studies because Branum et al. [33] focused on family groups to control for shared genetic or environmental factors. Two articles [42, 50] used data from the Bassett Mothers Health Project; given that both investigations focused on the same GWG exposures and outcomes (but at different ages), they were combined into a single study for analyses and interpretation.

### 3.3. GWG Measures

GWG is a composite variable that is comprised on measurements of prepregnancy weight, weight, and gestational age at delivery. Methods used to assess GWG varied considerably across the studies. As shown in Table 1, a majority of studies defined total GWG as the difference between mother's weight at delivery or near delivery and mother's prepregnancy weight [15]. Most of the included studies used the last weight measure during prenatal care visits but did not specify the mean duration of measurement time to delivery [23, 25, 31, 35, 38–40, 42, 47–50]. Four studies reporting this information differed in the proximity of last weight measurement prior to delivery (ranged from 37 weeks of gestation to just prior to delivery) which impacts their capacity to measure total weight gain throughout the whole pregnancy [33, 34, 43]. Additionally, one study measured weight within 12 hours after delivery, not accounting for the weight of fetus [37]. Two studies measured weight at 20th week and 30th week of gestation [24, 45]. Four studies asked women to recall their total GWG at postpartum [36, 41, 44, 46]. In terms of the measurement methods, most studies used self-reported prepregnancy weight or weight data abstracted from medical records. Only three studies used objectively measured weight in the early pregnancy [23, 37, 42].

GWG was used as either continuous and/or categorical variables. As a continuous variable, GWG was mainly coded in three ways: total GWG (*n* = 12) [25, 31–34, 36, 38, 39, 41, 44, 46, 47], net GWG (*n* = 5) [25, 37, 42, 48, 50], and rate of GWG (kg or lbs/week) (*n* = 4) [23, 32, 39, 40]. Total GWG is defined as the difference between mother's weight at delivery or near delivery and her prepregnancy weight. Net GWG was calculated by subtracting infant's birth weight from the total GWG, and this accounts for the variation in infant's birth weight. Due to the variation on the timing of weight gain measurements obtained during pregnancy as well as the differences in gestational age at delivery, some studies used the weekly rate of GWG. Weekly rate of GWG is defined as total GWG divided by the duration of pregnancy, expressed as weeks of gestation for the interval such as a trimester or at the visit [39]. Two studies used GWG at 20th week [24] and 30th week [45] of gestation as the exposure. Nine studies adopted the IOM guideline (either 1990 [25, 33, 35, 41, 43, 47] or 2009 [23, 34, 49] guidelines) to categorize maternal total GWG as inadequate, adequate, or excessive GWG. Additionally, two studies analyzed maternal total GWG as a categorical variable using arbitrary cut-off points [38, 46].

### 3.4. Child Body Weight Measures

Child body weight outcome was expressed as BMI *z*-score (continuous) in 10 studies and overweight status (categorical) in 13 studies. All studies from USA (*n* = 16) followed the CDC 2000 cut-off points [25, 31, 33, 35–37, 39–42, 47–50]. Five out of the seven European studies [23, 24, 32, 34, 45] used cut-off points from IOTF/WHO growth chart; one study [44] determined the cut-off points based on national growth chart and two studies [37, 46] used BMI (weight (kg)/height (m)^2^) as the outcome variable. In terms of measurement, 15 studies used objectively measured child body weight [23–25, 33, 34, 36–40, 43, 44, 46, 47, 49], four studies used self-reported [35, 41, 42, 50], and three studies used other anthropometrical measures (i.e., parental-reported [32, 45] or clinically recorded [48]). Only four studies included two or more measurement time points during the entire follow-up period [38, 42, 44, 49] and the remaining studies measured child's body weight once (Table 1).

#### 3.4.1. Methodological Quality Assessment

Three studies [23, 37, 48] were rated as having high methodological quality and 20 studies with medium quality [24, 25, 31–36, 38–47, 49, 50]. Overall, studies did not meet the high quality category because of the use of self-reported measures on GWG and child's body weight outcomes (Table 2).

#### 3.4.2. Total GWG and Offspring's Body Weight Outcomes

Table 3 summarized the strength of associations between various GWG measures and body weight outcomes in offspring. Seven out of eight studies [25, 32, 34, 39, 41, 44, 47] that examined the association between continuous total GWG and offspring's body weight outcomes found a significant positive association. That is, an additional kilogram increase in total GWG increased child's BMI *z*-score by 0.006 to 0.06 units and elevated the risk of overweight or obesity by 1% to 23% after adjusting for potential confounders (Table 3). Five studies [25, 31, 34, 36, 47] conducted stratified analyses to investigate the modifying effect of prepregnancy BMI on the association between total GWG and child's body weight outcomes (Table 4). One study [36] found that the direct effect of GWG on offspring's BMI *z*-score was stronger than indirect effects in normal-weight and overweight mothers.

Two studies used an arbitrary cut-off point to classify total GWG. Li et al. [38] examined total GWG in relation to the latent growth trajectory in offspring from age 2 to 12. The odds of having child with early-onset of overweight in mothers who gained ≥20.43 kg during pregnancy was 1.7 times that of mothers who gained between 11.35 and 15.88 kg (i.e., higher probability of being overweight between ages 2 and 6). However, total GWG was not associated with the late-onset of overweight in offspring (i.e., lower probability of overweight after 8 years of age). Stuebe et al. [46] categorized total GWG into <10, 10–14, 15–19, 20–29, 30–39, and ≥40 lbs. Their findings indicated a U-shape association between total GWG and offspring's weight status. Using mothers who gained 15–19 lbs as a reference group, the risk of overweight at age 18 significantly increased in offspring of mothers who gained <10 lbs (adjusted odds ratio (AOR): 1.51, 95% CI: 1.00–2.30), 10–14 lbs (AOR: 1.56, 95% CI: 1.13–2.16), and ≥40 lbs (AOR: 1.68, 95% CI: 1.13–2.52).

#### 3.4.3. Net GWG and Offspring's Body Weight Outcomes

Less evidence exists for an association between net GWG and child's body weight outcomes. Four studies [25, 31, 37, 48] demonstrated a positive relationship between net GWG and offspring body weight outcomes, three of which achieved statistical significance [25, 37, 48]. Increments in net GWG were associated with 0.01 to 0.07 unit increase in children's BMI *z*-score (Table 3).

The effect of maternal prepregnancy BMI on the association between net GWG and offspring's body weight outcomes was examined in one study. Lawlor et al. [37] found that, in the between-family model (participants from different families), the positive association between net GWG and offspring BMI at 18 years of age was stronger in normal-weight mothers than overweight mothers. In the within-family model (siblings from the same family), the positive association was retained in overweight mothers but not in normal-weight mothers (Table 4).

#### 3.4.4. Rate of GWG and Offspring's Body Weight Outcomes

Four studies [23, 32, 39, 40] investigated the association between rate of GWG and offspring's body weight outcomes. Although the calculation of rate of GWG varied among studies, these studies consistently demonstrated that high rate of GWG in early- and mid-pregnancy was associated with increased BMI *z*-score and elevated risk of overweight risk among offspring, while a null association was observed between rate of GWG at late pregnancy and child's body weight outcomes (Tables 3 and 4).

#### 3.4.5. IOM Recommended GWG and Offspring's Body Weight Outcomes

The evidence for an association between excessive GWG and offspring body weight outcomes was less than and not as consistent as total GWG. Eight studies [23, 31, 33, 34, 41, 43, 47, 48] compared the effects of excessive GWG versus adequate GWG on child's body weight outcomes, six of which achieved statistical significance [23, 31, 34, 41, 43, 47]. Offspring born to mothers who gained excessive weight during pregnancy had increased BMI *z*-scores (0.14 to 0.64 units) and elevated risks of overweight or obesity (27% to 73%) compared to offspring whose mothers gained adequate weight (Table 3).

Three studies [25, 35, 49] compared the effects of excessive GWG on offspring's risk of overweight with a different referent group. Lindberg and colleagues [49] compared the effects of excessive GWG and nonexcessive GWG (adequate GWG plus inadequate GWG) on offspring's risk of overweight between 5 and 8 years of age. The child's risk of overweight was 73% higher in children exposed to excessive GWG than those who did not. Two studies used data from Project Viva. Gillman et al. [35] compared the effects of excessive GWG versus nonexcessive GWG on offspring's risk of overweight and found a null association. Oken et al. [25] found that children exposed to excessive GWG had higher BMI *z*-score (0.47 units) and elevated risk of overweight (4-fold) than children exposed to inadequate GWG.

Eight studies [23, 31, 33, 34, 41, 43, 47, 48] showed mixed findings while examining the association between inadequate GWG and offspring body weight outcomes. Five studies [31, 33, 34, 43, 47] found a null association; three studies [23, 41, 48] found a negative association (0.06 to 0.21 units reductions in child's BMI *z*-score) (Table 3). Two studies [34, 47] conducted stratified analyses and found that the effects of excessive GWG on offspring's body weight outcome did not vary by maternal prepregnancy BMI (Table 4).

#### 3.4.6. Other GWG Measures and Offspring's Body Weight Outcomes

Laitinen et al. [24] found that an additional kilogram increase in total GWG during the first 20 weeks of pregnancy increased offspring's odds of developing overweight by 3%. Stamnes Køpp and colleagues [45] showed that total GWG at 30 weeks of gestation was associated with 0.02 unit increments in offspring's BMI at age 3.

## 4. Discussion 

This systematic review presents a summary of existing evidence on the associations of maternal weight gain during pregnancy with offspring body weight outcomes between 2 and 18.9 years from observational cohort studies. Overall, 23 studies met our inclusion criteria. Consistent with previous reviews [21, 22], we also found that higher total GWG significantly increased BMI *z*-score (0.006 to 0.06 units) and increased risk of overweight or obesity (1% to 23%). Compared to offspring whose mothers gained adequate weight during pregnancy, children of mothers who gained excessive weight had significantly higher BMI *z*-score (0.74 to 1.73 units) and elevated risk of overweight or obesity (1% to 57%).

A new finding in the present review is the potential impact of rate of GWG on offspring's body weight outcomes. Although an insufficient number of studies (*n* = 4) are available to draw a conclusion, they consistently demonstrated that high rates of GWG in early- and mid-pregnancy had strong adverse effects on offspring body weight outcomes. The underlying mechanisms regarding this association remain to be defined. Andersen and colleagues [32] performed path analyses and confirmed a direct pathway from rates of GWG in the early- and mid-pregnancy to offspring's body weight outcomes. We speculate that high rates of GWG in early- and mid-pregnancy increased maternal fat deposition and may have altered intrauterine environment for the development of fetal adipose tissues. Theoretically, maternal GWG can affect fetal adiposity accumulation in two possible pathways. The first one is direct transfusion of free fatty acids from the mother to fetus [52]. For underweight and normal weight women (prepregnancy BMI < 25 kg/m^2^), GWG in the early- and mid-pregnancy is disproportionately fat [53]. The fat mainly deposits in mother's hips, back, and upper thighs as a caloric reserve for late pregnancy and lactation [53]. Meanwhile, mid-pregnancy is recognized as a critical period when fetal fat tissue begins to grow [54, 55]. High rates of GWG in early- and mid-gestational periods could lead to excessive maternal fat deposition that may increase the transmission of free fatty acid from mother to fetus. The second pathway is the synthesis of free fatty acids from substrates such as glucose provided by the mother [52]. Excessive fat deposition during early pregnancy could reduce maternal insulin sensitivity and glucose tolerance [56, 57] to a greater extent than the normal metabolic sequelae of pregnancy. This loss of metabolic control could translate into elevated maternal glucose concentration (i.e., glycemic excursions) which exposes the fetus to an increased glucose supply [56, 57]. Both increased transfusion of lipid and increased supply of glucose from the mother may alter the development of fat cells in fetus, thus resulting in a permanent increase in fetus's capacity to form new cells in adipose depots in postnatal life [54, 58]. However, intensive studies are needed to test these speculations.

The current findings should be interpreted with caution due to several methodological concerns. One notable methodological concern is the failure to adjust for shared familial characteristics. In this review, only two studies employed a between- and within-family design to control for shared familial characteristics. Branum et al. [33] found that the significant association between total GWG and child's BMI *z*-score became nonsignificant after adjusting for the shared familial characteristics. These results indicated that the positive association between maternal total GWG and offspring's BMI *z*-score may be entirely due to shared genetics and environmental (e.g., family lifestyle) factors rather than the intrauterine environment. Lawlor et al. [37] found that the significant association disappeared in normal-weight mother but it remained significant in overweight mothers. These findings implied that, in normal-weight mother, the association between net GWG and offspring BMI is largely due to shared familial risk factors, whereas the association in the children of overweight and/or obese mothers is driven by the exposure to both familial characteristics and intrauterine environment. A recently published study [59] examined the independent effects of GWG on offspring body weight outcomes at 11.9 years of age in 42,133 women and their 91,045 offspring, using a within-family design to minimize confounding effects of shared familial characteristics. The results showed that total GWG significantly increased offspring's BMI *z*-score by 0.022 units and elevated their risk of overweight by 0.7% at 11 years of age. When classifying total GWG into categories (<6 kg, ≥12 to ≤18, and >18) variable, offspring BMI increased by 0.43 units and the risk of overweight or obesity increased by 8% when comparing children born to mothers who gained >18 kg during pregnancy to those whose mothers gained <6 kg. These associations were independent of child birth weight and other covariates (e.g., gestational age, maternal smoking, parity, child age, child BMI measured at earlier ages, etc.). These findings confirmed that, after adjusting for familial characteristics, overnutrition in pregnancy could program the fetus for an increased lifetime risk for overweight or obesity, though the magnitude of this effect may be small. Additionally, these studies also demonstrated that introducing shared familiar characteristics into the analyses significantly attenuated the magnitude of associations between GWG and offspring's body weight outcomes. Thus, this important confounding variable needs to be measured and adjusted in future studies.

Shared familial characteristics consist of both genetics and/or environmental factors such as lifestyle. Since none of the studies in our review has adjusted genetic factors as covariates, we are not able to examine its modifying effects on the association of interest. Lifestyle factors such as offspring's physical activity are consistently shown to be a significant predictor of the development of childhood obesity [60]. Besides, there is a strong correlation between maternal lifestyle and offspring behaviors [61, 62]. Recent research has suggested that maternal lifestyles have dramatically changed over the last half century. Maternal activity has decreased significantly over the past 50 years, with a concomitant increase in sedentary behaviors [63, 64]. Additionally, maternal self-reported dietary consumption of away-from-home foods (e.g., packaged and convenience foods like frozen pizza) [65], numbers of eating occasions, and portion sizes per eating occasion have increased significantly over the last 30 years [66]. These changes may have significant effects on childhood lifestyle behaviors such as physical activity, dietary behaviors, and consequent obesity. In the current study, only four studies [25, 34, 36, 41] controlled for child's lifestyle factors (i.e., subjectively measured physical activity and consumptions of unhealthy foods) in the analyses, and these studies found that these factors did not alter the association between GWG and child's body weight outcomes. However, the null association could be due to the attenuation induced via poor measurement (e.g., self-report measures tend to overestimate physical activity and underestimate intake of unhealthy food in children). More research is needed to verify which shared familial characteristics are influential to the association between maternal GWG and offspring body weight outcomes.

Additionally, none of the included studies has reported whether the study is powered to detect expected difference on the primary outcome and interactive effects by maternal prepregnancy BMI. By focusing exclusively or predominantly on Caucasian women, well-educated women, and nonobese women, the extant literature is not generalizable to high risk population such as African Americans, lower income, and overweight and obese women who are more likely to exceed weight gain recommendations during pregnancy than their counterparts [16, 67–69].

*Strengths and Limitations.* Compared to recently published meta-analyses [21, 22], our review has several strengths such as its focus on cohort studies and careful methodological examination of published studies in terms of quality and timing of GWG measurements, adjustment of confounding variables, statistical analyses, and associated interpretations. As with any study, this review has limitations. Publication bias may be presented as the current review only included English language and published peer-reviewed journal articles. The heterogeneity in the study samples, exposures, and outcome measures included in this review limited the interpretation of the evidence and prevented the use of meta-analytical methods. The semiquantitative reporting in this review provides only an arbitrary classification of the associations and focuses more on the direction of association rather than magnitude. Several studies have drawn data from the same cohort studies, for example, the Project Viva or National Collaborative Perinatal Project, which may introduce the issue of overrepresentation and bias into the analysis sample.

## 5. Conclusions

The current findings suggest that GWG is a potential risk factor to prevent childhood obesity. Additionally, GWG appears to be more strongly associated with offspring's body weight outcomes during early- and mid-pregnancy than late-pregnancy, and future studies are encouraged to examine the critical timing in which GWG had the strongest impact on child's body weight outcomes. Future research should also consider the following issues: adjusting confounding effects of shared familial characteristics, improving quality of the measurement on maternal prepregnancy weight, examining the underlying mechanism or pathways, and quantifying the impact among high risk population such as African American, obese, and low income women.

## Supplementary Material

Supplementary Table 1. Transparent Reporting of Systematic Reviews and Meta-analysis (PRISMA) checklist.
Supplementary Table 2. Systematic review search strategies.

## Figures and Tables

**Table 1:** Cohort studies on maternal weight gain during pregnancy and offspring's body weight outcomes.

Authors, year, country, and study design	Sample size and time period	Child age at follow-up	Definition of gestational weight gain (GWG)	GWG variable	Child body weight measure	Confounders adjusted
Li et al. 2007, USA (mixed) [38]	1,739 (1986–2000)	2–12 years	Self-reported weight before delivery minus self-reported prepregnancy weight	Total GWG (kg) (i) <6.8 (ii) 6.81–11.34 (iii) 11.35–15.88 (ref) (iv) 15.89–20.42 (v) ≥20.43	BMI *z*-score based on measured height and weight Early-onset OW: BMI ≥95th PCTL persisted from 2 to 8 years Late-onset OW: BMI ≥95th PCTL starting at 8 years (CDC)	Maternal age, alcohol consumption during pregnancy, education, family net income0 and prepregnancy BMI, and smoking during pregnancy; child's birth order, birth weight, birth year, breastfeeding, gestational age, race, and sex

Oken et al. 2007, USA (pros) [25]	1,044 (1999–2002)	3 years	Medical record retrieved last prenatal weight minus self-reported prepregnancy weight	Total GWG (kg) Net GWG (total GWG minus infant birth weight) IOM 1990 (i) Excessive (ii) Adequate (iii) Inadequate (ref)	BMI *z*-score based on measured height and weight OW: BMI ≥95th PCTL (CDC)	Mother's glucose tolerance, marital status, prepregnancy BMI, SES, smoking, and race; paternal BMI; and child breastfeeding duration, cesarean section, daily television viewing time, fast food and sugar beverage intake, gestational fetal growth, gestational length, and sex

Gillman et al. 2008, USA (pros) [35]	1,110 (1999–2002)	3 years	Medical record retrieved last prenatal weight minus prepregnancy weight	IOM 1990 (i) Excessive (ii) Nonexcessive (Ref)	BMI *z*-score based on self-reported height and weight OW: BMI ≥95th PCTL (CDC)	Maternal education, prepregnancy BMI, smoking during pregnancy, and SES; child breastfeeding duration, daily sleep during infancy, and race

Oken et al. 2008, USA (pros) [41]	11,994 (1996–1999)	9–14 years	Self-reported total GWG	Total GWG (lbs) IOM 1990 (i) Excessive (ii) Adequate (Ref) (iii) Inadequate	BMI *z*-score based on self-reported height and weight OW: BMI 85th to ≤95th PCTL OB: BMI >95th PCTL (CDC)	Maternal age, education, SES, gestational diabetes, marital status, prepregnancy BMI, smoking, and paternal education; and child age in 1996, birth weight, breastfeeding, daily sugar sweetened beverage intake, fried food away from home, gestational age, maturity, hours of TV and video, physical activity, and race

Wrotniak et al. 2008, USA (pros) [47]	10,226 (1959–1965)	7 years	Weight measured at delivery minus self-reported prepregnancy weight	Total GWG (kg) IOM 1990 (i) Excessive (ii) Adequate (Ref) (iii) Inadequate	BMI *z*-score based on measured height and weight OW: BMI ≥95th PCTL (CDC)	Maternal age, prepregnancy BMI, parity, race, and smoking; child age at 7-year assessment, birth weight, gestational age, and sex

Oken et al. 2009, USA (pros) [40]	2,012 (1999–2002)	3 years	Medical record retrieved last prenatal weight minus self-reported prepregnancy weight	Rate of GWG (kg/week)	BM *z*-score based on measured height and weight OB: BMI >95th PCTL (CDC)	Prepregnancy BMI

Olson et al. 2009, 2010, USA (pros) [42, 50]	321 (1997-1998)	4 years	Measured weight at last prenatal visit minus first measured weight in the first trimester	Net GWG (kg)	BMI *z*-score based on self-reported height and weight OW: BMI 85th to <95th PCTL OB: BMI ≥95th PCTL (CDC)	Maternal overweight at early pregnancy, SES, smoking during pregnancy, and SES; child birth weight, breastfeeding for at least 6 months, and gestational age

Stuebe et al. 2009, USA (mixed) [46]	26,506 (1989–2001)	7 years	Self-reported total GWG	Total GWG (lbs) (i) <10 (ii) 10–14 (iii) 15–19 (Ref) (iv) 20–29 (v) 30–40 (vi) >40	BMI based on measured height and weight OW: BMI ≥25 to <30 kg/m^2^ OB: BMI >30 kg/m^2^ (CDC)	Maternal age at child birth, prepregnancy BMI, maternal age at child's birth, nausea and smoking during pregnancy, family history of diabetes, parental BMI and education level, and mother living with father at time of child's birth; child birth weight and birth order

Fraser et al. 2010, UK (pros) [23]	5,154 (1991-1992)	9 years	Measured weight at last prenatal visit minus measured weight at first prenatal visit	Rates of GWG (g/week) IOM 2009 (i) Excessive (ii) Adequate (Ref) (iii) Inadequate	BMI based on measured height and weight (IOTF)	Maternal age, delivery mode, parity, prepregnancy BMI, parity, smoking during pregnancy, SES, and GWG in the previous pregnancy; child birth weight and gestational age

Magerison Zilko et al. 2010, USA (retro) [31]	4496 (1972–2000)	2–20 years	Self-reported weight at delivery minus self-reported prepregnancy weight	Total GWG (kg) IOM (2009) (i) Excessive (ii) Adequate (Ref) (iii) Inadequate	BMI *z*-score based on parent-reported or measured height and weight OW: BMI ≥85th PCTL (CDC)	Maternal age, education, poverty status, length of gestation, prepregnancy BMI, and race, smoking during pregnancy; child sex and year of birth; and weighted for sampling proportion

Schack-Nielsen et al. 2010, Denmark (pros) [44]	4,234 (1959–1961)	1–14 years	Self-reported total GWG	Total GWG (kg) (i) <6 (ii) 6–8 (iii) 9–10 (iv) 11-12 (v) 13–15 (vi) ≥16 kg The values of 5.5, 7.0, 9.5, 11.5, 14.0, and 16.5 were assigned mid-points to get continuous GWG	BMI *z*-score based on measured height and weight (British 1990 growth chart)	Maternal age, edema during prepregnancy, marital status, SES, and smoking during pregnancy; parental education, prepregnancy BMI, and prematurity; and child birth weight, gestational age, and sex

Andersen et al. 2011, Denmark (pros) [32]	9,869 (1996–2002)	7 years	Self-reported total GWG	Total GWG rates of GWG [kg in early (12–20 weeks) and mid-pregnancy (25–32 weeks)]	BMI *z*-score based on parent-reported height and weight (IOTF)	Maternal age, parity, smoking during pregnancy, prepregnancy BMI, SES, and paternal BMI; child age, birth weight, breastfeeding, gestational age, weight at 5 and 12 months, and sex

Branum et al. 2011, USA (pros) [33]	5,917 (1959–1965)	4 years	Measured weight at last prenatal visit within 3 wk of delivery minus self-reported prepregnancy weight	Total GWG IOM 1990 (i) Excessive (ii) Adequate (Ref) (iii) Inadequate	BMI *z*-score based on measured height and weight (CDC)	Maternal age, parity, prepregnancy BMI, race, SES, and smoking; child birth weight, gestational age, and sex

Lawlor et al. 2011, Sweden (mixed) [37]	14,6894 (1973–2005)	18 years	Measured weight within 12 h after delivery minus the first antenatal clinic assessment (~10 wk gestation)	Net GWG (kg)	BMI based on measured height and weight (CDC)	Maternal age, education, gestational diabetes, parity, and early-pregnancy BMI; child birth weight, gestational age, and year of birth

Ronney et al. 2011, USA (retro) [43]	450 (1988)	4-5 years	Measured weight prior to delivery minus self-reported prepregnancy weight	IOM 1990 (i) Excessive (ii) Adequate (Ref) (iii) Inadequate	BMI *z*-score based on measured height and weight OB: ≥85th PCTL	Maternal marital status, GWG in first 4 months and smoking, during pregnancy; child insurance status at birth and sex

Ensenauer et al. 2013, Germany (mixed) [34]	6,837 (2009–2011)	5 years	Measured weight at an average of 38-wk of gestation minus measured prepregnancy weight	Total GWG IOM 2009 (i) Excessive (ii) Adequate (Ref) (iii) Inadequate	BMI *z*-score based on measured height and weight OW: BMI ≥90th PCTL. OB: BMI ≥97th PCTL (IOTF)	Maternal age and smoking during pregnancy; child age, birth weight, breastfeeding, TV viewing, physical activity, and SES

Hinkle et al. 2012, USA (mixed) [36]	3,600 (2001–2006)	5 years	Total GWG from birth certificates (81%) plus maternal report (19%) at 9-month postpartum	Total GWG (kg)	BMI *z*-score based on measured height and weight OW: BMI 85th to <95th PCTL. OB: BMI ≥95th PCTL (CDC)	Maternal age, race, parity, marital status, education, participation in special supplement nutrition program for women and child, smoking at the last 3 months of pregnancy, and postpartum exercise habits; child's exercise habit; and child birth weight, breastfeeding, gestational age, sugar-sweetened beverage intake, fast food intake, and TV viewing

Laitinen et al. 2012, Finland (pros) [24]	6,637 (1985–2002)	16 years	Measured weight at 20-wk gestation minus self-reported prepregnancy weight	GWG at 20-week gestation (kg) (quartiles were used)	BMI *z*-score based on measured height and weight (IOTF)	Maternal education, glucose metabolism, hemoglobin at 8–10 weeks of gestation, parity, prepregnancy BMI, and smoking; child's sex

Linberg et al. 2012, USA (mixed) [49]	471 (2004)	5–8 years	Medical record retrieved weight at delivery minus recorded prepregnancy weight	IOM 2009 (i) Excessive (ii) Not excessive (Ref)	BMI *z*-score based on measured height and weight OW: BMI ≥85th PCTL (CDC)	Maternal education, gestational diabetes, prepregnancy BMI, and smoking before and during pregnancy; child's birth weight and breastfeeding duration

Magerison-Zilko et al. 2012, USA (pros) [39]	3,015 (1959–1967)	5 years	Medical record retrieved last prenatal weight minus self-reported prepregnancy weight	Total GWG trimester-specific rates of GWG	BMI *z*-score based on measured height and weight OW: BMI ≥85th PCTL (CDC)	Maternal age, education, marital status, parity, prepregnancy BMI, smoking during pregnancy, and race; paternal overweight; and child gestational age, and sex

Stamnes Køpp et al. 2012, Norway (pros) [45]	5,898 (1999–2009)	3 years	Self-reported weight at 30-week pregnancy minus self-reported prepregnancy weight	GWG at 30-week gestation (kg)	BMI based on parent-reported height and weight	Maternal age, education, exercise habit, parity, prepregnancy BMI, smoking during pregnancy, and paternal BMI; child birth weight, breastfeeding at 6 month, types of day care, hours in screen-based activities, and sex

Ehrenthal et al. 2013, USA (retro) [48]	3,320 (2004–2007)	4 years	Self-reported weight at delivery minus self-reported prepregnancy weight	Net GWG (adjusted for gestational age) (kg) IOM 2009 (i) Excessive (ii) Adequate (Ref) (iii) Inadequate	BMI *z*-score based on medical record retrieved height and weight (CDC)	Maternal age, gestational diabetes and hypertension, insurance status, marital status, parity, preeclampsia, prepregnancy BMI, diabetes, hypertension, race, and smoking during pregnancy; child age and child born to same mother

BMI = body mass index; CDC = Center for Disease Control and Prevention; GWG = gestational weight gain; IOM = Institute of Medicine; IOTF = International Obesity Task Force; mixed = mixed cohort; NW = normal weight; OB = obese; OW = overweight; PCTL = percentile; pros = prospective cohort; Ref = referent group; retro = retrospective cohort; SES = socioeconomic status; UW = underweight; and WHO = World Health Organization.

**Table 2:** Methodological quality assessment of included cohort studies.

	Li et al. [38]	Oken, et al. 2007 [25]	Gillman et al. [35]	Oken, et al. 2008 [41]	Wrotniak et al. [47]	Oken, et al. 2009 [40]	Olson et al. [42, 50]	Steube et al. [46]	Fraser et al. [23]	Magerison Zilko et al. [31]	Schack-Nielsen et al. [44]	Andersen et al. [32]	Branum et al. [33]	Lawlor et al. [37]	Rooney et al. [43]	Ensenauer et al. [34]	Hinkle et al. [36]	Laitinen et al. [24]	Lindberg et al. [49]	Magerison-Zilko et al. [39]	Stamens-Køpp et al. [45]	Ehrenthal et al. [48]
**(1) Description of background ** Presented in context of previous research, hypothesis clearly described. *✓*: 2 elements, O: 1 element, and X: 0 element presented	*✓*	*✓*	*✓*	*✓*	*✓*	*✓*	*✓*	*✓*	*✓*	*✓*	*✓*	*✓*	*✓*	*✓*	*✓*	*✓*	*✓*	*✓*	*✓*	*✓*	*✓*	*✓*

**(2) Sample definition** Explicit inclusion/exclusion criteria, uniform application of criteria, clear description of recruitment strategy and participant's characteristics, power analysis, or some other basis noted for determining the adequacy of study sample size. *✓*: >3 or more elements, O: 2 or 3 elements, and X: <2 elements presented	*✓*	*✓*	*✓*	*✓*	*✓*	*✓*	*✓*	*✓*	*✓*	*✓*	*✓*	*✓*	*✓*	*✓*	*✓*	*✓*	*✓*	*✓*	*✓*	*✓*	*✓*	*✓*

**(3) Description of gestational weight gain** Clearly described prepregnancy weight and prenatal weight. *✓*: ≥2 elements, O: 1 element, and X: 0 element details described	*✓*	O	*✓*	*✓*	*✓*	O	O	X	*✓*	*✓*	O	*✓*	O	*✓*	*✓*	*✓*	*✓*	*✓*	X	O	X	*✓*

**(4) Description of child body weight** Clearly defined child body weight outcome and described definition for overweight/obesity. *✓*: ≥2 elements presented, O: moderately or very clear definition of weight gain, and X: poor definition of child body weight outcome	*✓*	*✓*	*✓*	*✓*	*✓*	*✓*	*✓*	*✓*	*✓*	*✓*	*✓*	*✓*	*✓*	*✓*	*✓*	*✓*	*✓*	*✓*	*✓*	*✓*	*✓*	*✓*

**(5) Soundness of information on GWG.** Quality of source of information on GWG *✓*: objective measures, O: self-report with validation, and X: Self-report	X	X	X	X	X	X	*✓*	X	*✓*	O	X	X	X	*✓*	X	X	X	X	X	X	X	O

**(6) Soundness of information** Quality of source of information on child body weight outcome. *✓*: objective measures, O: parental-reported, and X: self-report	*✓*	*✓*	X	X	*✓*	*✓*	X	*✓*	*✓*	O	*✓*	O	*✓*	*✓*	*✓*	*✓*	*✓*	*✓*	*✓*	*✓*	O	*✓*

**(7) Description on reduction of the final sample** Adequate reporting on loss to follow-up and the number of participants at each stage of study. *✓*: both elements, O: 1 element, and X: 0 elements details described	*✓*	*✓*	*✓*	*✓*	*✓*	*✓*	*✓*	*✓*	*✓*	*✓*	*✓*	*✓*	*✓*	*✓*	*✓*	*✓*	*✓*	*✓*	*✓*	*✓*	*✓*	*✓*

**(8) Analysis comparability** Adequately accounted for withdrawals, lost to follow-up, and missing data in the analysis, appropriate statistical methods were used for main analysis and adjustment of potential confounders. *✓*: all elements clearly presented, O: some presented, and X: neither element present	*✓*	*✓*	*✓*	*✓*	*✓*	O	*✓*	*✓*	*✓*	*✓*	*✓*	*✓*	*✓*	*✓*	*✓*	*✓*	*✓*	*✓*	*✓*	*✓*	*✓*	*✓*

**(9) Interpretation of results** Results interpreted appropriately based on study design and statistics, clinically useful, appropriate presentation, present in the context of prior research, and conclusion supported by results. *✓*: all elements clearly present, O: any other score, and X: conclusion not supported by results	*✓*	*✓*	*✓*	*✓*	*✓*	O	*✓*	*✓*	*✓*	*✓*	*✓*	*✓*	*✓*	*✓*	*✓*	*✓*	*✓*	*✓*	*✓*	*✓*	*✓*	*✓*

**Overall quality** (i) High (H): ≥ 6 good rating (*✓*) AND zero poor rating (X) (ii) Medium (M): <6 good ratings (*✓*) OR ≥6 good ratings (*✓*) and ≤2 poor ratings (X) (iii) Low (L): ≥3 poor ratings (X) OR any other score	M	M	M	M	M	M	M	M	**H**	M	M	M	M	**H**	M	M	M	M	M	M	M	**H**

Notes: *✓* = good; O = fair; and X = poor.

**Table 3:** Summary of the association between maternal GWG and offspring body weight outcomes.

Study	Child age	Child BMI *z*-score Beta coefficient	Child OW/OB status ARR or AOR
Total GWG^#^			
Oken et al. 2007 [25]	3	0.06 (0.05, 0.07)	OW: 1.23 (1.16, 1.30)
Branum et al. 2011 [33]	4	Within-family: −0.03 (−0.08, 0.02) Between-family: 0.01 (−0.02, 0.04)	
Ensenauer et al. 2013 [34]	5.8		OW: 1.04 (1.02, 1.05)
Magerison-Zilko et al. 2012 [39]	5	0.02 (0.01, 0.03)	OW: 1.04 (1.02, 1.07)
Andersen et al. 2011 [32]^a^	7	0.04 (0.03, 0.06)	
Wrotniak et al. 2008 [47]	7		OW: 1.03 (1.01, 1.05)
Schack-Nielsen et al. 2010 [44]	1–14	0.01 to 0.03 (NA)	
Oken et al. 2008 [41]	9–14	0.006 (0.005, 0.007)	OW: 1.05 (1.04, 1.05) OB: 1.08 (1.07, 1.08)
Net GWG			
Olson et al. 2009 [42, 50]	3		OW: 1.001 (NS) OB: 1.010 (NS)
Oken et al. 2007 [25]	3	0.02 (0.01, 0.03)	
Ehrenthal et al. 2013 [48]	4	0.012 (0.006, 0.017)	
Rate of GWG			
Magerison-Zilko et al. 2012 [39]^b^	5		OW: Early: 1.05 (1.02, 1.09) Mid: 1.03 (0.98, 1.08) Late: 1.03 (0.98, 1.08)
Andersen et al. 2011 [32]^c^	7	Early: 0.05 (0.03, 0.07) Mid: 0.06 (0.04, 0.08) Late: 0.016 (−0.002, 0.03)	
Fraser et al. 2010 [23]^d,e^	9	Early/low rate: 0.17 (−0.20, 0.53) Early/medium rate: 0.33 (0.11, 0.55) Early/high rate: 0.62 (0.24, 1.01)	OW: Early/low rate: 1.06 (0.77, 1.47) Early/medium rate: 1.14 (0.92, 1.42) Early/high rate: 1.57 (1.13, 2.18)
		Mid/low rate: −0.54 (2.06, 0.99) Mid/medium rate: 0.39 (−0.07, 0.84) Mid/high rate: 0.62 (0.26, 0.99)	Mid/low rate: 1.05 (0.28, 4.00) Mid/medium rate: 0.98 (0.62, 1.54) Mid/high rate: 2.00 (1.43, 2.79)
		Late/low rate: 0.091 (−0.35, 0.53) Late/medium rate: −0.031 (−0.48, 0.42) Late/high rate: 0.17 (−0.13, 0.46)	Late/low rate: 0.88 (0.57, 1.36) Late/medium rate: 1.02 (0.64, 1.61) Late/high rate: 1.06 (0.81, 1.39)
Excessive GWG^¶^			
Branum et al. 2011 [33]	4	Within-family: 0.01 (−0.13, 0.14) Between-family: 0.01 (−0.08, 0.10)	
Ehrenthal et al. 2013 [48]	4	0.051 (−0.039, 0.140)	
Ensenauer et al. 2013 [34]	5.8		OW: 1.57 (1.30, 1.91)
Wrotniak et al. 2008 [47]	7		OW: 1.40 (1.00, 1.95)
Fraser et al. 2010 [23]	9	0.64 (0.55, 0.94)	
Magerison Zilko et al. 2010 [31]	2–20		OW: 1.27 (1.10, 1.48)
Oken et al. 2008 [41]	9–14	0.14 (0.09, 0.18)	OW: 1.27 (1.12, 1.44) OB: 1.42 (1.19, 1.70)
Rooney et al. 2011 [43]	9–14		OB: 1.73 (1.06, 2.80)
Inadequate GWG			
Branum et al. 2011 [33]	4	Within-family: 0.08 (0.00, 0.16) Between-family: 0.04 (−0.02, 0.10)	
Ehrenthal et al. 2013 [48]	4	−0.190 (−0.319, −0.062)	
Ensenauer et al. 2013 [34]	5.8		OW: 1.20 (0.91, 1.57)
Wrotniak et al. 2008 [47]	7		OW: 0.93 (0.72, 1.21)
Fraser et al. 2010 [23]	9	−0.21 (−0.40, −0.03)	
Magerison Zilko et al. 2010 [31]	2–20		OW: 0.90 (NS)
Oken et al. 2008 [41]	9–14	−0.06 (−0.10, −0.01)	OW: 0.97 (1.19, 1.70) OB: 0.91 (0.74, 1.13)
Rooney et al. 2011 [43]	9–14		OB: 0.77 (0.45, 1.34)

^a^log transformed value.

^b^Rate of GWG expressed as change in kilograms per trimester. Early: 1st trimester, mid: 2nd trimester, and late: 3rd trimester.

^c^Rate of GWG expressed as change in grams per week. Early: until interview 1 (12–20 weeks of gestation), mid: between interview 1 and interview 2 (25–32 weeks of gestation), and late: between interview 2 and delivery.

^d^Rate of GWG expressed as change in grams per week. Early: 0–14 weeks of gestation, mid: >14–35 weeks of gestation, and late: >36 weeks of gestation; low rate: ≤0 g in 0–14 weeks of gestation, ≤250 g per week in other GWG periods, medium rate: 0–500 g in 0–14 weeks of gestation, 250–500 g in other GWG periods, and high rate: >500 g for all GWG period.

^e^BMI (kg/m^2^) was used as the outcome.

^#^Only studies that used total GWG as continuous variables and presented full sample analyses are included.

^¶^Only studies that used adequate GWG as the referent group are included.

ARR = adjusted relative risk, AOR = adjusted odd ratio, GWG = gestational weight gain, NA = not available, NS = not significant, OW = overweight, and OB = obesity.

**Table 4:** Summary of the association between maternal GWG and offspring's body weight outcomes stratified by maternal prepregnancy BMI.

Study	Child age	Child BMI *z*-score, B coefficient (95% CI)				Child OW/OB status, AOR/ARR (95% CI)			
		UW	NW	OW	OB	UW	NW	OW	OB
Total GWG									
Oken et al. 2007 [25]	3		0.02 (0.02, 0.06)	0.03 (0.02, 0.04)					
Hinkle et al. 2012 [36]	5	−0.06 (−0.16, 0.03)	0.02 (0.00, 0.04)	0.02 (−0.01, 0.06)	0.00 (−0.04, 0.03)				
Ensenauer et al. 2013 [34]	5.8					1.03 (0.90, 1.16)	1.04 (1.02, 1.07)	1.01 (0.98, 1.04)	1.04 (1.01, 1.07)
Wrotniak et al. 2008 [47]	7						1.07 (0.99, 1.15)	1.01 (0.99, 1.03)	
Magerison Zilko et al. 2010 [31]	2–20					1.02 (0.99, 1.05)	1.03 (1.02, 1.04)	1.02 (1.00, 1.04)	1.02 (1.00, 1.04)
Net GWG									
Lawlor et al. 2011 [37]^a^	18		Within-family: 0.01 (−0.02, 0.02) Between-family: 0.07 (0.06, 0.07)	Within-family: 0.06 (0.01, 0.02) Between-family: 0.02 (0.01, 0.03)					
Rate of GWG									
Oken et al. 2009 [40]^b^	3						1.16 (0.88, 1.51)	1.35 (1.01, 1.81)	1.22 (0.96, 1.56)
Excessive GWG									
Ensenauer et al. 2013 [34]	5.8					1.50 (0.36, 6.39)	1.29 (1.01, 1.66)	1.64 (1.06, 2.63)	1.17 (0.70, 2.01)
Wrotniak et al. 2008 [47]	7						3.26 (0.95, 11.16)	1.48 (1.05, 2.08)	
Inadequate GWG									
Ensenauer et al. 2013 [34]	5.8					1.74 (0.30, 8.97)	1.02 (0.71, 1.43)	2.52 (1.28, 4.91)	0.63 (0.30, 1.30)
Wrotniak et al. 2008 [47]	7						0.55 (0.22, 1.21)	0.95 (0.73, 1.24)	

^a^BMI (kg/m^2^) was used as the outcome.

^b^Rate of GWG expressed as change per 0.1 kg per week.

GWG = gestational weight gain, UW = underweight, NW = normal weight, OW = overweight, OB = obesity, 95%, and CI = 95% confidence interval.
